# Proteomic Adaptation of *Clostridioides difficile* to Treatment with the Antimicrobial Peptide Nisin

**DOI:** 10.3390/cells10020372

**Published:** 2021-02-11

**Authors:** Sandra Maaß, Jürgen Bartel, Pierre-Alexander Mücke, Rabea Schlüter, Thomas Sura, Julia Zaschke-Kriesche, Sander H. J. Smits, Dörte Becher

**Affiliations:** 1 Department of Microbial Proteomics, Institute of Microbiology, University of Greifswald, Felix-Hausdorff-Str. 8, 17489 Greifswald, Germany; bartelj@uni-greifswald.de (J.B.); pierre.muecke@uni-greifswald.de (P.-A.M.); thomas.sura@uni-greifswald.de (T.S.); dbecher@uni-greifswald.de (D.B.); 2 Imaging Center of the Department of Biology, University of Greifswald, Friedrich-Ludwig-Jahn-Str. 15, 17489 Greifswald, Germany; rabea.schlueter@uni-greifswald.de; 3 Department of Chemistry, Institute of Biochemistry, Heinrich-Heine University Düsseldorf, Universitätsstr. 1, 40225 Düsseldorf, Germany; julia.schumacher@hhu.de (J.Z.-K.); sander.smits@uni-duesseldorf.de (S.H.J.S.)

**Keywords:** nisin, antimicrobial peptides, antibiotic resistance, mass spectrometry, *C. difficile*, proteomics

## Abstract

*Clostridioides difficile* is the leading cause of antibiotic-associated diarrhea but can also result in more serious, life-threatening conditions. The incidence of *C. difficile* infections in hospitals is increasing, both in frequency and severity, and antibiotic-resistant *C. difficile* strains are advancing. Against this background antimicrobial peptides (AMPs) are an interesting alternative to classic antibiotics. Information on the effects of AMPs on *C. difficile* will not only enhance the knowledge for possible biomedical application but may also provide insights into mechanisms of *C. difficile* to adapt or counteract AMPs. This study applies state-of-the-art mass spectrometry methods to quantitatively investigate the proteomic response of *C. difficile* 630∆*erm* to sublethal concentrations of the AMP nisin allowing to follow the cellular stress adaptation in a time-resolved manner. The results do not only point at a heavy reorganization of the cellular envelope but also resulted in pronounced changes in central cellular processes such as carbohydrate metabolism. Further, the number of flagella per cell was increased during the adaptation process. The potential involvement of flagella in nisin adaptation was supported by a more resistant phenotype exhibited by a non-motile but hyper-flagellated mutant.

## 1. Introduction

Lantibiotics are ribosomally synthesized antimicrobial peptides produced by a range of Gram-positive bacteria [1]. During the synthesis of lantibiotics, posttranslational modifications lead to the creation of unusual amino acids such as dehydroalanine, dehydrobutyrine or lanthionine. In fact, the resulting lanthionine residues were not only eponymous (from *lanthionine* containing ant*ibiotics*) for this important class of bacteriocins but also ensure the high antimicrobial activity of lantibiotics against various, mainly Gram-positive, bacteria. This high activity in combination with the high stability against proteolytic digestion facilitated the usage of lantibiotics as food preservatives. Moreover, it has frequently been suggested to use the great potential of lantibiotics in a wide range of medical applications [2,3,4].

Besides well-known lantibiotics such as gallidermin and subtilin, nisin is probably the best characterized one. The cationic, linear peptide of 34 amino acids is naturally produced by *Lactococcus lactis* subsp*. lactis* and contains five stereo-specifically installed lanthionine-rings. Nisin was first marketed in England in 1953 to inhibit the outgrowth of *Clostridium tyrobutyricum,* which is responsible for late cheese blowing and leads to food-spoiling [5]. Due to its high bactericidal activity in combination with low toxicity in humans [6], nisin has been applied for decades in the dairy and food industries to prevent the growth of pathogenic bacteria contaminating food. To date, nisin is the only bacteriocin approved as a food preservative. It has been shown that nisin also exhibits antimicrobial effects on a range of nonfood-related bacteria such as Methicillin-resistant *Staphylococcus aureus* (MRSA), Vancomycin-resistant enterococci (VRE), and *Streptococcus pneumoniae* [7,8,9]. Hence nisin has been recognized as possible treatment option for those pathogens.

Against Gram-positive bacteria nisin has at least a dual mode of action as it disturbs cell wall biosynthesis and also forms pores in the cell membranes of susceptible cells [10]. After initial electrostatic attraction of the cationic peptide by the anionic envelope of target bacteria, rings A and B of nisin bind to N-acetylmuramic acid, the isoprene unit of lipid II [11,12], and undecaprenyl pyrophosphate, thereby sequestering those cell wall precursors into stable complexes [13,14]. This is in contrast to the binding of the well-known glycopeptide antibiotics vancomycin and teicoplanin, which recognize the D-Ala-D-alanyl group of lipid II [15]. The lantibiotic–lipid II complex subsequently inhibits peptidoglycan synthesis by physically sequestering lipid II and preventing its utilization by penicillin-binding proteins. Moreover, the formed complex facilitates pore formation by rings D and E of nisin, which are able to flip into the bacterial cell membrane leading to the release of ions and small molecules from the target bacteria [10,16,17]. Indeed treatment of *Clostridioides difficile* vegetative cells with nisin was associated with significant holes in the cell membrane [18].

*C. difficile* is an anaerobic, spore-forming bacterium that is one of the most ubiquitous nosocomial pathogens. *C. difficile* infection (CDI) is the leading cause of antibiotics-associated diarrhea [19] but can also result in more serious, life-threatening conditions such as pseudomembranous colitis, toxic megacolon, and intestinal perforation [20,21]. Incidence of CDI in hospitals is increasing, both in frequency and severity, resulting in considerable morbidity and mortality and hence representing an enormous financial burden for the health care system [22].

Bacterial resistance to nisin can be mediated by a range of general mechanisms including alterations to the surface charge or changes in the composition of the membrane as well as the formation of spores or biofilms. D-alanylation of teichoic acids in the cell wall of Gram-positive bacteria including *C. difficile* will result in the addition of positive charges to the cell surface [23,24] leading to the repulsion of cationic antimicrobial peptides from the cell surface. The D-alanine esterification of teichoic acids is catalyzed by four proteins encoded in the *dlt* (D-alanyl-lipoteichoic acid) operon: DltA, DltB, DltC, and DltD [23]. All four proteins are required for the successful addition of D-alanine to the cell wall [24,25] and it has been shown that the expression of the *dlt* operon increases resistance to AMPs such as nisin [24,25].

Adaptation to nisin may also result in changes in the phospholipid composition of the bacterial cell membrane. The switch from “negatively-charged” phosphatidylglycerol and cardiolipin to zwitterionic phosphatidylethanolamine and phosphatidylcholine decreases the overall negative charge at the membrane and hence potentially diminishes the ability of nisin to interact with the cell envelope [26]. Moreover, “nisin-resistant” cells have been reported to exhibit lower levels of phospholipids [27], a higher percentage of straight-chain fatty acids and a lower percentage of branched-chain fatty acids than the parental cells [28]. Reduced membrane fluidity due to increased long-chain fatty acids and reduced ratios of C15/C17 fatty acids has also been associated with nisin resistance [29]. Additionally, the presence of aminoacylated phosphatidylglycerols imparting a net positive charge on the cell surface provides a way for some bacteria to shield themselves from the action of lantibiotics. The protein catalyzing this aminoacylation, MprF, has been described for *S. aureus* [30] and some other Gram-positive bacteria, such as *Clostridium perfringens,* which possess homologous genes. However, no homologue could be found in *C. difficile* 630.

In *C. difficile* the *cpr* (cationic antimicrobial peptide resistance) system contributes to lantibiotic resistance. In this system the sensor kinase CprK and an orphan response regulator, CprR, are responsible for the resistance to cationic antimicrobial peptides, including nisin [31]. CprK and CprR are both expressed at low levels prior to lantibiotic exposure, but CprK expression is markedly induced upon activation by nisin, while CprR expression is not [31]. The two-component system CprKR regulates the expression of genes encoding for the ABC transporter CprABC, which has naturally been shown to be induced after the addition of nisin [31]. Additionally, adjacent to the *cprABC* gene cluster, a lipoprotein is present, which is not upregulated by the presence of a lantibiotic or antimicrobial peptide and displays a basal expression level [31,32].

As recent research has shown that the antimicrobial activity of nisin not only applies to food-contaminating pathogens but also extends to a range of non-food related bacteria [7,8,9], nisin has been recognized as a possible treatment option against those pathogens. In such applications, nisin may, due to its insensitivity to degradation by the components of the jejunal chime [33], also enter the large intestine of patients, where it may come in contact with *C. difficile*. Moreover, nisin shares physicochemical properties with host defense peptides such as LL-37, which play an essential role in the innate immune response [34,35]. Indeed there is evidence that nisin influences the immune system of mice [36] and is able to activate neutrophils [37]. Hence nisin might also have effects on other subsets of host immune cells. The idea that nisin behaves similarly to host defense peptides, might explain the findings that nisin is able to promote synergistic effects in combination with conventional antibiotics [8,38] and shows beneficial properties during the treatment of bacterial infections of the skin, gastrointestinal and respiratory tract [34]. On the other hand, actively growing *C. difficile* must contend with host defense peptides in the intestinal environment. Knowledge on the effects of nisin on *C. difficile* will therefore not only enhance the knowledge for possible biomedical applications of lantibiotics but may also provide insights in mechanisms of *C. difficile* to adapt or counteract a number of innate immune factors produced by either host cells [39] or by the intestinal microflora [40].

The current study uses state-of-the-art mass spectrometry methods to quantitatively investigate the proteomic response of *C. difficile* 630∆*erm* to sublethal concentrations of nisin allowing to follow the cellular effects of stress imposition and during adaptation to nisin in a time-resolved manner. In order to discriminate between changes in the cytosolic and the membrane protein fraction, both sub-proteomes were prepared from the same samples and analyzed in parallel. The results point towards a drastic reorganization in both the membrane-associated and the soluble sub-proteome thereby hinting at early stress responses that are different from the already described physiology observed in long-term adapted or even resistant cells.

## 2. Materials and Methods

### 2.1. Purification of Nisin

Nisin was purified with an ion-exchange chromatography as previously described [41] and the concentration determined with RP-HPLC as published elsewhere [42].

### 2.2. Bacterial Growth and Nisin Treatment for Proteomic Analysis

**Figure 1:**
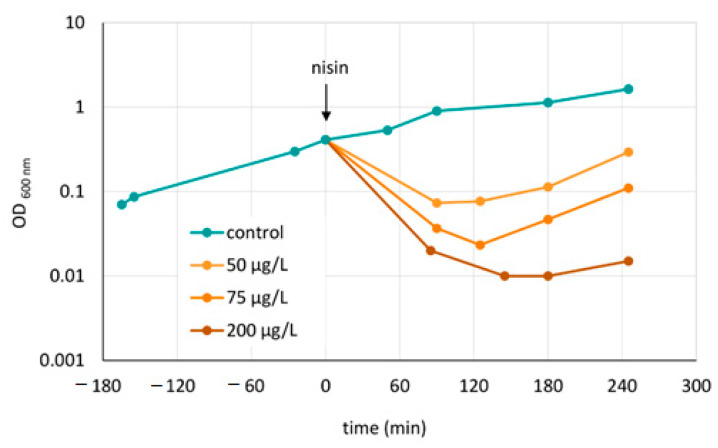
Growth of *C. difficile* 630∆*erm* after treatment with nisin. Cell culture was performed at 37 °C in brain heart infusion (BHI) in an anaerobic chamber. At OD_600 nm_ = 0.4 (exponential growth phase, corresponding to 4.06 ∗ 10^7^ ± 7.84 ∗ 10^6^ cfu/mL) cultures were either treated with nisin (shades of orange) or left untreated (control, blue). Samples were taken before the treatment (time = 0 min, control), 90 min and 180 min after addition of 50 µg/L nisin.

*C. difficile* 630∆*erm* [43], here named wild type, was grown at 37 °C in brain heart infusion (BHI) in an anaerobic chamber. Experiments were performed in three biological replicates. To generate those replicates, cells from three independent stationary cultures (prepared from spores) were passaged to fresh medium and cultivated for at least 16 h. Those three overnight cultures were used to inoculate three main cultures representing the three biological replicates. In the exponential growth phase (OD_600 nm_ of 0.4) cultures were stressed with sublethal concentrations of nisin (50 µg/L), resulting in a rapid decrease of optical density and subsequent re-growing with a growth rate comparable to an untreated control culture (Figure 1). Samples were taken before the treatment (control), 90 min, and 180 min after the addition of nisin.

To support our hypothesis on the role of flagellar during adaptation to nisin, a *sigH* mutant strain, overexpressing most of the genes of the flagellum locus [44], was grown and treated as the wild type and growth was monitored at OD_600 nm_.

### 2.3. Sample Preparation for Mass Spectrometry

Cells were harvested before the treatment (control), 90 min, and 180 min after addition of nisin by centrifugation and were subsequently resuspended in TE-buffer (10 mM Tris, 5 mM EDTA [ethylenediaminetetraacetic acid], pH 7.5). Cells were lysed by ultrasonication at 4 °C (Sonoplus HD 3200 [BANDELIN electronic, Berlin, Germany] with MS73, 3 cycles of 1 min 70% amplitude). Cell debris was removed by centrifugation (10 min, 9000× *g*, 4 °C) and the supernatant was used to determine the protein concentration with the Bradford assay [45] before 4 mg protein were used to enrich membrane proteins according to a protocol published elsewhere [46], leaving out the extraction of proteins by n-dodecyl-β-d-maltoside treatment. In brief, sedimented cell membranes were subjected to subsequent ultracentrifugation/washing steps, including washing of the pellets with high-salt and carbonate buffer resulting in the membrane fraction that is depleted of cytosolic proteins. The cytosolic fraction represents the supernatant after the first ultracentrifugation step in the membrane-enrichment protocol. Twenty µg of protein samples were supplemented with loading buffer (final concentration: 0.1 M Tris-HCl [pH 6.8], 2% [*w*/*v*] SDS, 10% [*v*/*v*] glycerol, 2% [*v*/*v*] β-mercaptoethanol), heated for 5 min at 95 °C, and separated via SDS-PAGE (Criterion TG 4–20% Precast Midi Gel, BIO-RAD Laboratories, Inc., Hercules, CA, USA). After staining with Coomassie, each gel lane was cut into pieces, destained, desiccated, and rehydrated in trypsin as previously described [47]. In gel-digest was incubated at 37 °C overnight. Peptides were eluted with water by sonication for 15 min and desalted using Millipore C18 Zip Tips (Sigma Aldrich, Taufkirchen, Germany) according to the manufacturer’s protocol.

### 2.4. Liquid Chromatography and Mass Spectrometric Analysis

Peptides derived from the cytosolic fraction were loaded on an EASY-nLC II system (Thermo Fisher Scientific, Dreieich, Germany) equipped with an in-house built 20 cm column (inner diameter 100 µm, outer diameter 360 µm) filled with ReproSil-Pur 120 C18-AQ reversed-phase material (3 µm particles, Dr. Maisch GmbH, Ammerbuch-Entringen, Germany). Elution of peptides was executed with a nonlinear 80 min gradient from 1 to 99% solvent B (0.1% (*v*/*v*) acetic acid in acetonitrile) with a flow rate of 300 nL/min and injected online into an LTQ Orbitrap XL (Thermo Fisher Scientific). The survey scan at a resolution of R = 30,000 and 1 × 10^6^ automatic gain control target in the Orbitrap with activated lock mass correction was followed by a selection of the five most abundant precursor ions for fragmentation. Singly charged ions, as well as ions without detected charge states, were excluded from MS/MS analysis.

Peptides derived from the membrane fraction were loaded on an EASY-nLC 1000 system (Thermo Fisher Scientific) equipped with the same in-house built column as described above. Elution of peptides was executed with the same gradient described above and injected online into an Orbitrap Q Exactive (Thermo Fisher Scientific). The survey scan at a resolution of R = 70,000 at *m*/*z* 200 and 3 × 10^6^ automatic gain control target in the Orbitrap with activated lock mass correction was followed by a selection of the 10 most abundant precursor ions for fragmentation. Single charged ions, as well as ions without detected charge states, were again excluded from MS/MS analysis.

All MS data were deposited to the ProteomeXchange Consortium via the PRIDE partner repository [48] with the dataset identifier PXD021684.

### 2.5. Data Processing and Data Analysis

Identification of peptides was carried out by database search using MaxQuant 1.5.8.3 with the implemented Andromeda algorithm [49] applying the following parameters: digestion mode, trypsin/P with up to 2 missed cleavages; variable modification, methionine oxidation, and maximal number of 5 modifications per peptide; activated LFQ option with minimal ratio count of 2 and “match-between runs” feature. The false discovery rates of peptide spectrum match and protein level were set to 0.01. Only unique peptides were used for protein quantification. The database for *C. difficile* 630∆*erm* [50] contained 3781 entries. Common laboratory contaminations and reverse entries were added during MaxQuant search. A protein was considered to be identified if two or more unique peptides were identified in a biological replicate.

The comparative proteome analyses of treated and control samples based on MaxQuant LFQ values were performed separately for cytosolic and membrane samples. Proteins were considered to be quantified if a quantitative value based on at least two unique peptides was available in at least two biological replicates. Log2-transformed LFQ values as proxy for protein abundance were used for statistical analysis. An ANOVA test was performed to analyze changes in protein amounts during nisin treatment (control vs. 90 min nisin vs. 180 min nisin). Additionally, Student’s *t*-tests were performed to test for significant changes between samples (control vs. 90 min nisin and 90 min nisin vs 180 min nisin, respectively). Proteins with significantly changed amount exhibited a *p*-value < 0.01 and an average log2-fold change >|0.8|. For comparison of absolute protein amounts in the membrane fraction iBAQ values [51] were exported from MaxQuant and width adjusted in Perseus 1.5.3.0 [52].

### 2.6. Electron Microscopy

To determine the number of flagella, nisin treated and non-treated wild type cells were negatively stained and examined with a transmission electron microscope LEO 906 (Carl Zeiss Microscopy GmbH, Jena, Germany) at an acceleration voltage of 80 kV as previously described [53]. For image acquisition, a wide-angle dual speed CCD camera Sharpeye (Tröndle, Moorenweis, Germany) was used, operated by the ImageSP software. Afterwards all micrographs were adjusted by using Adobe Photoshop CS6. Transmission electron micrographs at a 10.000-fold magnification of at least 10 cells per condition were used to determine the number of flagella. Statistical significance was assessed by an unpaired Student’s *t*-test.

## 3. Results

### 3.1. Phenotypic and Proteomic Adaptation

In order to find a nisin concentration that exhibits sublethal effects to *C. difficile* cells and hence allows for sampling of intact cells, exponentially growing *C. difficile* 630∆*erm* in BHI medium was treated with different amounts of nisin and the optical density was monitored for at least four hours after nisin addition (Figure 1). The growth curves and earlier determination of propidium iodide (PI) uptake [54] revealed rapid lysis of *C. difficile* cells after nisin addition that was dose-dependent. Moreover, it became obvious that there was a correlation between the extent of cell lysis and the time needed for the culture to restart growth.

Based on the results of the growth experiments 50 µg/L nisin were used for proteome analysis in order to apply an amount of stressor which still allows to harvest of a sufficient number of intact cells. Due to the biphasic characteristics of the culture after nisin addition, two sampling points, 90 and 180 min after nisin addition, were selected to cover both the phase of cell lysis and growth recovery (Figure 1).

Although nisin targets the cell envelope, basic metabolic pathways and fundamental cellular processes, which might be part of any adaptive response, are catalyzed by enzymes located in the cytosol. Hence, a separate analysis of cytosolic and membrane proteome is supposed to provide valuable information on protein abundances within the subcellular fractions and even on the relocalisation of proteins between both sub-proteomes. In order to discriminate between changes in the cytosolic and the membrane protein fraction, both sub-proteomes were prepared from the same samples and analyzed in parallel. In total 2055 proteins, representing 54% of the predicted proteome of *C. difficile* 630∆*erm*, could be identified in this study of which 1853 proteins could be quantified in at least two out of three biological replicates in any sample. In total 792 proteins exhibited significant changes in protein abundance in at least one of the sub-proteomes during adaptation to nisin pointing at a drastic reprogramming of protein expression in response to nisin treatment.

### 3.2. Adaptation in the Cytosol

As a considerable number of cellular processes are carried out by cytosolic proteins, the fraction of soluble proteins was analyzed to gain knowledge on the adaptation to nisin. In this study, 1084 proteins were identified from the cytosolic protein fraction of which 924 proteins were quantified. Out of those, 112 proteins exhibit a significant change in abundance after nisin addition. All quantitative data obtained from the cytosolic protein fraction can be found in Supplemental Table S1.

**Figure 2:**
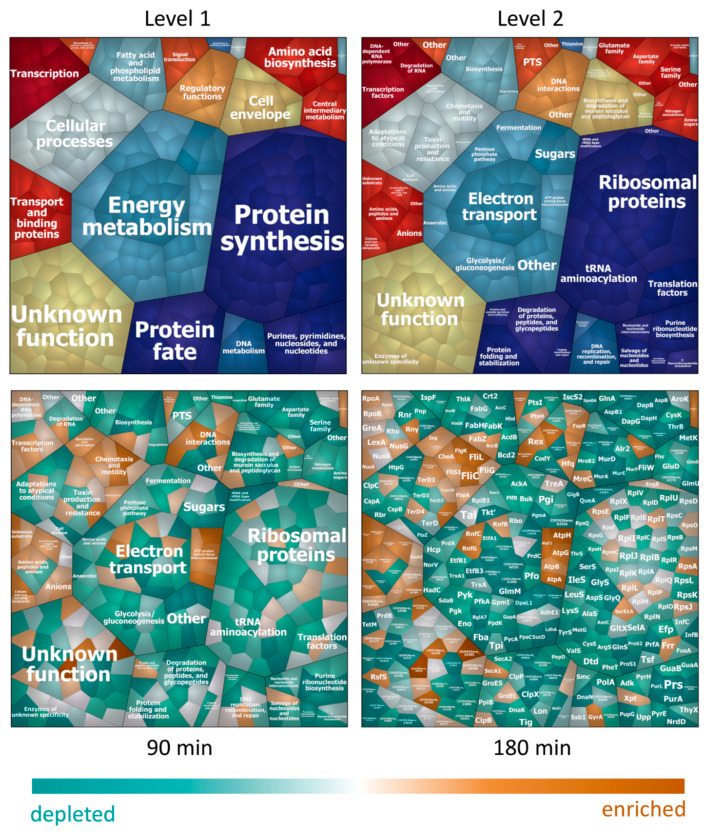
Voronoi Treemaps for proteins in the cytosolic fraction. Proteins predicted to be located in the cytosol or with unknown localization, which were quantified in the cytosolic fractions of all samples, are depicted in tiles and were hierarchically clustered according to TIGRFams. The upper part of the figure displays the first two cluster levels of assigned functions. The third level displaying protein names for every single tile is shown in the map for timepoint 180 min. A list of assigned functional groups to all proteins detected in the cytosolic fraction can be found in Supplemental Material (Supplemental Table S1). The lower part of the figures displays the relative protein abundance at 90 min (**left**) and 180 min (**right**) after nisin treatment compared to untreated control conditions, respectively. Tiles of proteins with higher abundance after nisin treatment are colored in shades of orange, tiles of proteins with lower abundance after treatment are colored in turquoise.

Some of the observed proteomic changes reflect the changing growth rates determined during phenotypic analysis performed in this study. Indeed, proteins involved in protein biosynthesis, especially ribosomal proteins and proteins involved in tRNA aminoacylation, decreased during treatment and only showed a slight increase in abundance again 180 min after nisin addition (Figure 2). As growth rate-dependent effects on the proteome have already been studied in detail [55,56], we focused our data analysis on proteomics adaptations associated with the AMP-treatment.

The second noticeable observation was the differential expression of many transcriptional regulators during adaptation to nisin. Most important in the context of antimicrobial resistance might be the role of regulators belonging to the MarR and GntR protein family. In *Escherichia coli*, MarR is a repressor, which regulates the expression of its own operon and genes for multi-drug efflux systems, thus conferring resistance to multiple antibiotics and other toxic compounds [57,58]. In the current study, the MarR family protein CDIF630erm_00954 was strongly (>20-fold) induced 180 min after nisin addition.

GntR family members fulfil various biological functions in diverse bacterial groups. Amongst others, GntR-like proteins are involved in resistance to antimicrobial compounds including vancomycin and isoniazid, which inhibit cell wall synthesis [59,60,61]. In this study, we found the GntR-like protein CDIF630erm_01007 with significantly altered amounts after the addition of nisin to *C. difficile*.

The third striking effect of nisin on *C. difficile* pertains to carbohydrate metabolism. Indeed, proteins involved in sugar transport as well as in anabolism and catabolism of various sugars were found in altered amounts during adaptation to nisin (Supplemental Tables S1 and S2). Moreover, the accumulation of σ^54^-dependent gene products could be observed. σ^54^ has been linked to transcription of genes involved in the catabolism of various carbohydrates including β-glucosides, fructose/levan, mannose/glucose, pentitols, and glucosamine/fructosamine in diverse Firmicutes species [62]. Besides the targets of the alternative sigma factor, also the sugar responsive regulators DeoR and RpiR accumulated up to 30-fold, of which the latter one is known to link the metabolic response with virulence in *Staphylococcus aureus* [63]. Moreover, the synthetic enzymes for N-acetylglucosamine and rhamnose, both part of *C. difficile’s* cell envelope, accumulated during nisin adaptation. Together, the data suggest a serious perturbation of *C. difficile’s* cell wall synthesis and, as various sugars are also needed to produce surface polysaccharides [64], the cell surface of the pathogen. This is not unexpected, as nisin targets the cell envelope. Hence, one may assume further adaptational mechanisms to be discovered when inspecting the membrane proteome in more detail.

### 3.3. Composition of the Membrane Proteome during Adaptation to Nisin

As nisin is known to attach to and disturb the bacterial envelope it can be expected that proteomics adaptations can also be observed in the membrane fraction as a consequence of the bacterial cell’s attempt to adapt the cell envelope to the stressor. In this study, 1970 proteins could be identified in this specific sub-proteome out of which 1768 proteins could be quantified (Supplemental Table S2). In total, 749 quantified proteins showed a significantly changed abundance during adaptation to nisin.

Although a clear enrichment of predicted membrane proteins can be observed, the fraction of proteins with a predicted localization in the cytosol is still very high (Supplemental Figure S1). This could be caused by a tight functional association of cytosolic proteins with the cellular membrane or the accumulation of misfolded proteins that aggregate in the membrane protein fraction. Thus, in order to conclude on the physiological processes in the cell membrane during nisin treatment, proteins with a predicted localization in or at the membrane were in the focus of the data analysis.

**Figure 3:**
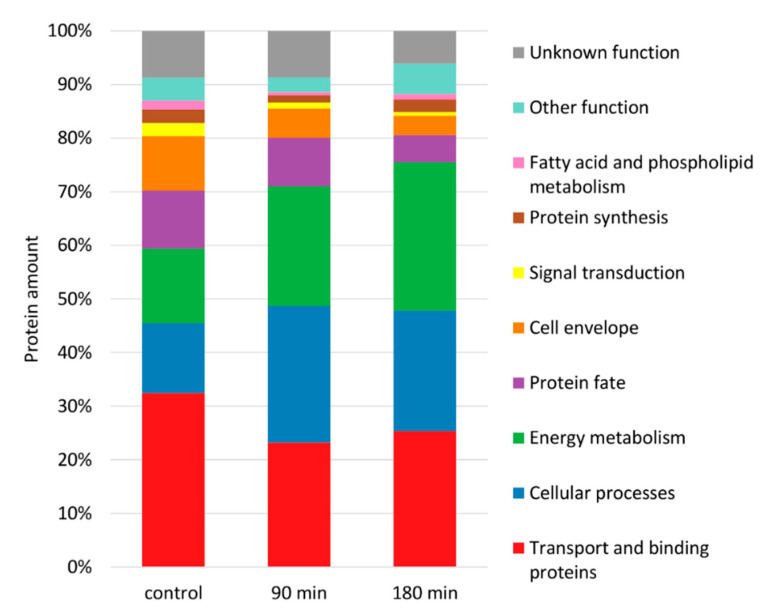
Changes in the composition of the membrane proteome after nisin treatment. The fraction of absolute protein amounts assigned to functional categories (TIGRFams, first level) was calculated by summing up iBAQ [51] intensities for all proteins quantified in the membrane fraction at the respective sampling point, which are predicted to be localized in the cell membrane, the cell wall or have an unknown localization. Only the highest abundant functional groups are shown. All other functional categories are summarized as “other function”.

**Figure 4:**
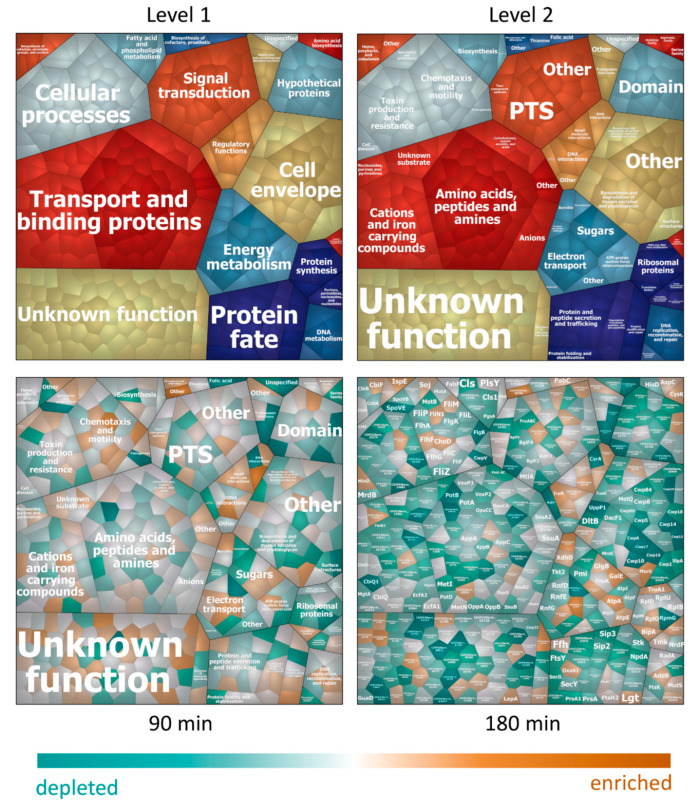
Voronoi Treemaps for proteins in the membrane fraction. Proteins predicted to be located in the cell membrane, the cell wall or with unknown localisation, which were quantified in the membrane fraction of all samples, are depicted in tiles and were hierarchically clustered according to TIGRFams. The upper part of the figure displays the first two cluster levels of assigned functions. The third level displaying protein names for every single tile is shown in the map for timepoint 180 min. A list of assigned functional groups to all proteins detected in the membrane fraction can be found in Supplemental Material (Supplemental Table S2). The lower part of the figures displays the relative protein abundance at 90 min (**left**) and 180 min (**right**) after nisin treatment compared to untreated control conditions, respectively. Tiles of proteins with higher abundance after nisin treatment are colored in shades of orange, tiles of proteins with lower abundance after treatment are colored in turquoise.

Experimental data on absolute protein amounts showed that transport and binding proteins were the most abundant proteins in the membrane followed by proteins involved in energy metabolism, various cellular process (e.g., chemotaxis and motility, cell division, pathogenesis), protein fate, and maintenance of the cell surface (Figure 3). Moreover, data revealed that proteins involved in shaping the cell envelope were depleted during nisin stress whereas enzymes providing energy were simultaneously enriched (Figure 3 and Figure 4). Interestingly transport and binding proteins showed a biphasic behavior being depleted during early phases of nisin treatment (90 min after nisin addition) and enriched again to nearly control level during adaptation to nisin (180 min after addition) (Figure 3). The opposite effect, an early enrichment (after 90 min of nisin stress) and a subsequent depletion of protein mass can be observed for proteins assigned to chemotaxis and motility (Figure 4).

**Figure 5:**
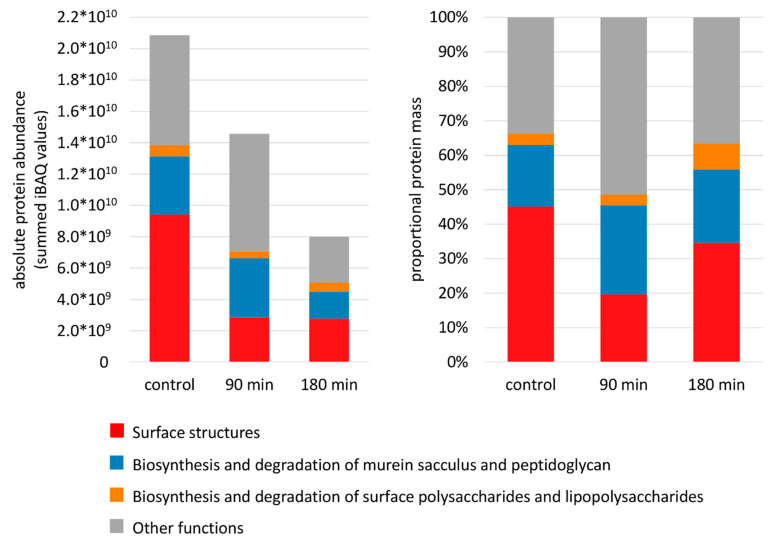
Changes in abundance of cell surface-associated proteins after nisin treatment. The fraction of proteins assigned to the cell surface was calculated by summing up iBAQ intensities for those proteins quantified in the membrane fraction at the respective sampling point. The figure compares the absolute protein amount provided as summed iBAQ values (**left**) to the proportional protein mass given in percent (**right**).

During in-depth analyses of proteins assigned to the different functional categories it was confirmed, that proteins assigned to cell surface function (e.g., structural surface proteins, proteins involved in the metabolism of the cell wall and surface polysaccharides) did not only show a decreased portion of the total membrane proteome after addition of nisin (Figure 3), but were also depleted in their absolute abundance (Figure 5, left). Moreover, it could be observed that the described quantitative effects can be mainly attributed to proteins providing cell surface structures such as SlpA, Cwp2, Cwp29 and the lipoprotein CDIF630erm_02274.

On the other hand, the fraction of proteins involved in peptidoglycan metabolism among all cell surface-associated proteins was enriched at 90 min after nisin addition (Figure 5, right). In particular, the amount of MurG, the central enzyme catalyzing the synthesis of lipid II from lipid I, was found to be increased up to 3.7-fold after nisin addition, showing the highest absolute abundance after 90 min of stress (Supplementary Table S2).

Other players modifying the cell envelope are the proteins encoded in the *dlt* operon, which confers resistance to cationic antimicrobial peptides, e.g., in *Bacillus subtilis* and *C. difficile*, by catalyzing the incorporation of D-alanine into teichoic acids [23,25]. Indeed it was shown, that the *dlt* operon is necessary for full resistance of *C. difficile* to nisin, gallidermin, polymyxin B and vancomycin [25]. While recent work describes an increase in *dltD* transcription during growth in the presence of nisin for 18 h [25], the metabolic proteins encoded by the *dlt* operon did not significantly accumulate within the 3 h after nisin addition examined in the current study. The different expression of the *dlt* operon in the two experimental setups ([25] and current study) points at differences between the early phase and fully established adaptation to nisin.

The third system expected to play a role in nisin adaptation and resistance development is the *cpr* operon. Indeed, expression of *cprABCKR* in *C. difficile* results in resistance against different lantibiotics including nisin, mutacin 1140, subtilin, and gallidermin, whereby only the sensorkinase CprK of the regulating two-component system CprKR is markedly induced upon activation by nisin, while CprR expression is not [31]. CprKR controls the expression of genes encoding for the ABC transporter CprABC, which have been shown to be induced after the addition of nisin [31]. CprA was also found in this proteomic study to accumulate up to 9-fold within the first 90 min after nisin treatment while returning to basal levels at 180 min after the addition of the lantibiotic. However, neither for CprK nor CprR changes in protein amounts could be detected in the experimental setup used here.

### 3.4. Relocalization of Proteins in Response to Nisin

Due to subcellular fractionation, changes in protein abundances could be quantified separately either in the cytosolic fraction, mainly representing soluble proteins, or in the membrane fraction, representing proteins connected to the cellular membrane, the cell wall as well as insoluble protein aggregates. Indeed, 124 proteins predicted to be located in the cell wall or associated with the membrane were detected in the cytosolic fraction after nisin treatment (Supplemental Figure S1). As the biosynthesis of membrane proteins takes place in the cytosol before their insertion into the membrane it is likely that those membrane proteins are detected in the soluble fraction. Moreover, only 29 membrane proteins detected in the cytosolic fraction obtain more than one predicted transmembrane helix, making their preparation together with soluble proteins even more reasonable. On the other hand, 960 cytosolic proteins were identified in the membrane fraction after nisin treatment (Supplemental Figure S2). The presence of cytosolic proteins in the membrane fraction may have different reasons: (i) the algorithms predicting their localization failed, (ii) they may have not been sufficiently removed during enrichment of the membrane fraction, (iii) they are co-purified with the membrane fraction because they are in a functional (or even physiological) complex with membrane-associated proteins, (iv) they aggregate in a denatured form due to irreversible protein damage or even degradation and accumulate in the membrane fraction after ultracentrifugation. Of note, 60 transcriptional regulators with predicted localization in the cytosol could be quantified in the membrane fraction after nisin treatment, which represents more than 60% of all regulators quantified in this study.

As various proteins are quantified in a subcellular fraction which did not match their predicted localization, the abundances of those proteins in either the membrane or the cytosol were examined in more detail. If the same proteins were identified in both sub-proteomes, changes in protein abundance were primarily observed in their predicted localization. For example, proteins involved in DNA metabolisms as well as in biosynthetic and catabolic pathways were found to be differentially abundant only in the soluble but not in the membrane fraction. This is not surprising as most metabolic processes are conducted in the cytosol.

Of the proteins found to be significantly regulated in both subcellular fractions, 70% showed a contrary regulation in the different sub-proteomes. Among them, there is a significant proportion of proteins that reflect the perturbed integrity of the cell surface such as proteins involved in the maintenance of the cell envelope, transport and binding proteins as well as proteins involved in chemotaxis and motility.

**Figure 6:**
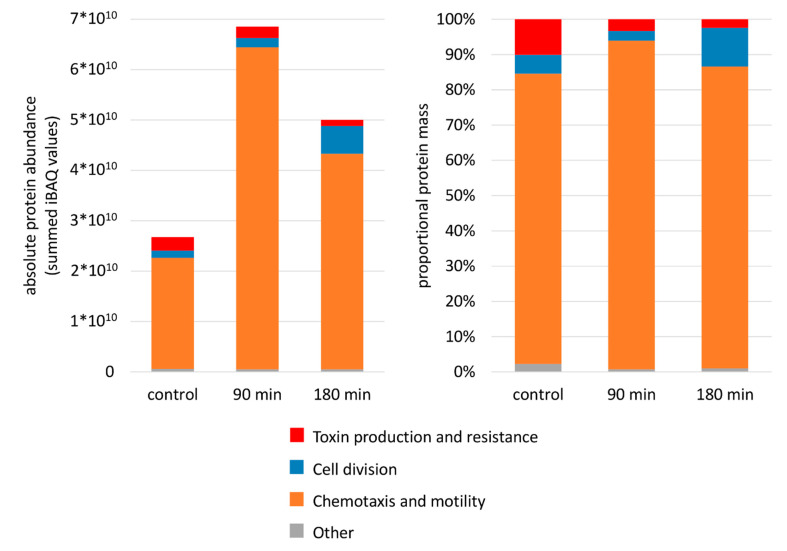
Changes in abundance of proteins involved in cellular processes (according to TIGRFams) after nisin treatment. The fraction of proteins assigned to this functional category was calculated by summing up iBAQ intensities for those proteins quantified in the membrane fraction at the respective sampling point. The figure compares the absolute protein amount provided as summed iBAQ values (**left**) to the proportional mass given in percent (**right**).

The analysis of protein relocalization during nisin treatment in *C. difficile* revealed the differential accumulation and depletion of flagella proteins in the different phases of adaptation. This already became obvious when biphasic changes in proteins amount were observed for the functional group of cellular processes, which includes proteins associated with toxin production, cell division, and cell motility (Figure 3). Indeed, the accumulation of those proteins in the early phases after nisin addition (90 min after nisin treatment) and their depletion during adaptation to nisin (180 min after addition) is mainly caused by flagella proteins assigned to the subcategory “chemotaxis and motility” (Figure 6).

**Figure 7:**
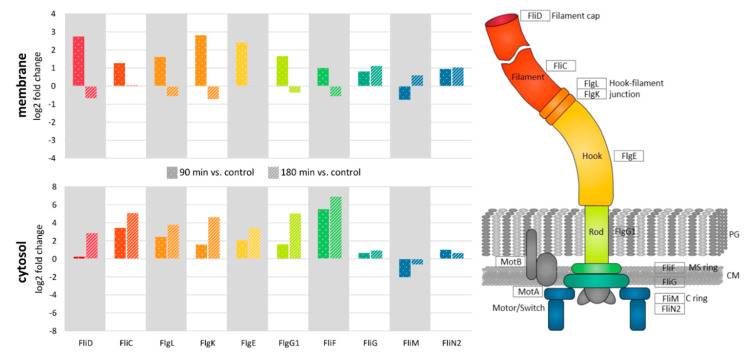
Relocalization of flagella proteins between cytosolic and membrane fraction after nisin treatment. The log2-fold change in protein abundance is depicted for flagella proteins quantified either in the cytosolic (“cytosol”) or the membrane fraction. Log2 ratios are calculated by comparing protein abundances 90 min (dotted bars) or 180 min (striped bars) after nisin treatment, respectively, to the respective protein abundance under control conditions (untreated).

A closer look at flagella proteins revealed their accumulation in the membrane fraction already at 90 min after nisin addition whereas at 180 min after nisin addition those proteins show an amount comparable to the amount in the untreated control (Figure 7). On the other hand, the same proteins accumulate in the cytosolic fraction only at 180 min after nisin treatment. The detection of flagella peptides in the soluble fraction simultaneous to a decreased flagella protein abundance in the membrane might hint at active disassembly or even degradation of structural flagella proteins in the later phase of nisin adaptation. Of note, the observed effects were stronger for the outer parts of the flagellum than for the membrane-anchored flagella structures positioned in the inner cell.

**Figure 8:**
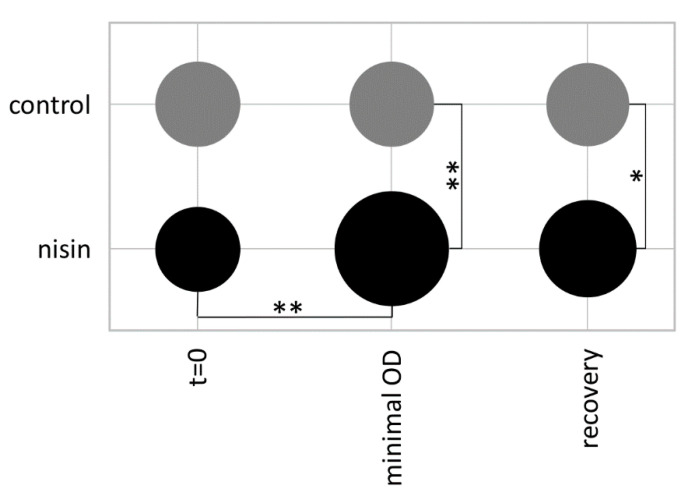
Number of flagella per cell of *C. difficile* 630∆*erm* during adaptation to nisin. The number of flagella was obtained from transmission electron micrographs and the average value for at least ten bacterial cells was used in this diagram (see Supplemental Figure S2 for representative images). Nisin was added to the cells displayed in line “nisin” whereas those displayed in line “control” were not treated. Samples were taken at the timepoint of nisin addition to the nisin culture (t = 0), when the culture reached the lowest OD (minimal OD) and during the phase of growth recovery (recovery) (see Figure 1 for a representative growth curve). Student’s *t*-tests were performed for every comparison along horizontal lines for “control” and “nisin” conditions to analyze statistical significance along the time series. Additional *t*-tests along vertical lines were performed to analyze statistical significance between “control” and “nisin” conditions at every timepoint. (*) indicates a *p*-value < 0.05, (**) indicates a *p*-value < 0.001.

As the data point at an increase in flagella proteins after nisin treatment, which is reversed to basal levels when the cells restart growth, electron microscopy was used to determine the number of flagella per cell. Indeed, the number of flagella per cell significantly increased from about 15 under non-stress conditions to 28 at the time point of the lowest optical density (see also Figure 1) and was reduced to about 21 flagella per cell during the phase of growth recovery (Figure 8).

**Figure 9:**
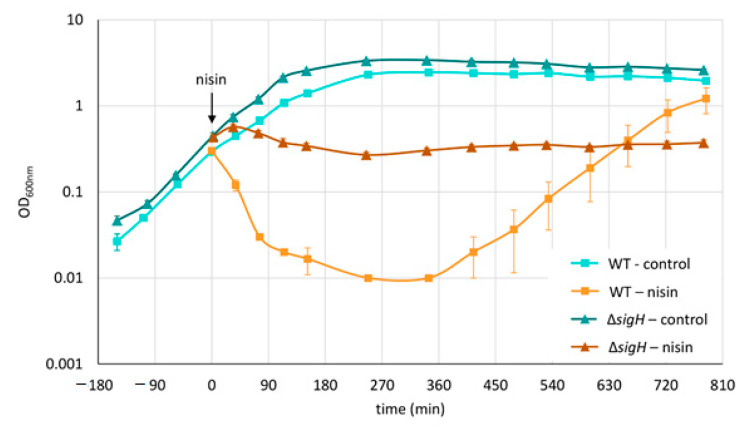
Growth of wild type and *sigH* mutant strain after treatment with nisin. *C. difficile* 630∆*erm* (WT, light colors, squares) and a *sigH* mutant strain (ΔsigH, dark colors, triangles) were grown at 37 °C in brain heart infusion (BHI) in an anaerobic chamber. In the exponential growth phase cultures were either treated with nisin (shades of orange) or left untreated (control, shades of blue). Error bars represent standard deviations of the optical densities determined in three biological replicates.

As the data suggested the involvement of flagella proteins in the adaptation to nisin, we next examined the growth of a *sigH* mutant strain in response to nisin. While the expression of most of the genes of the flagellum locus (43 genes) are upregulated in a *sigH* mutant, this mutant is non-motile in BHI [44,53]. In line with our hypothesis; the *sigH* mutant showed increased resistance to nisin when compared to the wild type (Figure 9).

## 4. Discussion

### 4.1. Differences between Early Phase and Fully Established Adaptation or Resistance

While recent work describes an increase in *dltD* transcription during growth in the presence of nisin for 18 h [25], the corresponding proteins did not significantly accumulate within the short time after nisin addition examined in the current study. Such divergent results could be potentially explained by differences in either, the applied antimicrobial concentration and the duration of treatment. Indeed, the existing literature points at different effects that can be observed when the same stressor is applied either in shock experiments (exponentially growing cells are treated with sub-lethal concentrations of the stressor) or in long-term stress experiment, where the bacterium is inoculated into a medium already containing the stressor and growth occurs in presence of this stressor for at least 16 h [53,61,65,66]. Moreover, the applicable concentration of a stressor seems to depend on the experimental setup followed. It was observed that in long-term stress experiments considerably higher amounts of the stressor can be applied compared to the sub-lethal concentrations used in corresponding shock experiments [53,66], which points at the ability of bacteria to adapt to the presence of any stressor given a sufficient amount of time. The fact that the minimal inhibitory concentration of nisin was determined to be 90 µg/mL in long-term stress setups [25] whereas we applied only 50 ng/mL in the current shock experiments supports this assumption. However, not only the concentration of the stressor differs in both experimental designs but also the observed cellular response seems to be dependent on the workflow followed. Steil et al. investigated the initial reaction of *B. subtilis* to a sudden rise in salinity and compared the results with the cellular adaptation to prolonged growth under high-salinity conditions [66]. The results of this study show that only a short, transient change in the expression pattern of genes was provoked by moderate salt shock while long-term salt stress had profound effects on the transcriptional profile. The probably best known representative for genes and proteins exhibiting only a transient response to stress is the alternative sigma factor SigB in *B. subtilis* for which a transient transcription of regulated genes and a short-term accumulation of target proteins have been described [67,68,69]. The current study shows that there is also a short-term adaptation in the abundance of other proteins. For example, the abundance of flagellar proteins changed in the first 90 min after nisin addition and was detected near their basal level at 180 min after stress. Besides the short-term adaptations observed in a shock experiment the study of Steil et al. also showed that only a small portion of the genes, that are immediately regulated by the shock, also displayed significant differences in cells harvested after long-term stress [66]. A comparable observation was made when *C. difficile* was treated with bile acids in both shock and long-term stress experiments [53], where, in contrast to the shock experiments, no general bile acids stress response was detectable after long-term treatment. Taken together the differences in the expression of the *C. difficile dlt* operon and its corresponding proteins in response to nisin may also be explained by the varying experimental setups applied.

### 4.2. Role of Flagella in Virulence and Resistance

This study revealed an increased abundance of flagellar proteins in the first 90 min after nisin addition followed by a relocalization of flagella subunits to the cytosol after 180 min (Figure 7), which might hint at an active disassembly of flagella after the initial adaptation to the applied lantibiotic. In line with that, the number of flagella per cell was increased when nisin-treated cultures reached their minimal OD and showed almost basal levels after recovery of growth (Figure 8). Hence, it is tempting to speculate that accumulation of flagella might increase resistance to nisin by (i) enhancing cell mobility enabling to physically escape the stressor, (ii) binding nisin and hence preventing its interaction with lipid II and incorporation into cell membranes or (iii) physically shielding the cell’s surface from nisin by forming capsular-like structures. Indeed, the role of *C. difficile* flagella in virulence and pathogenesis is described as contradictory in literature and seems to be dependent on the strain used. That said, all published studies coincide that flagella-mediated motility might contribute to the overall fitness of the bacteria. However, as the heavily flagellated but immotile *sigH* mutant showed increased resistance to nisin when compared to the wild type strain (Figure 9), the positive effect of increasing the number of flagella during nisin adaptation cannot be justified by enhanced cell mobility, which makes the second and third hypothesis or a combination thereof more likely.

The observed differential abundance of proteins involved in sugar metabolism can also be linked to the observed changes in flagellation. Three genera of *Clostridium*, including *C. difficile*, have been shown to glycosylate their flagellins [70,71]. In *C. difficile* 630 the specific glycosyltransferase (CD0240, CDIF630erm_00362) has been identified to be involved in the transfer of N-acetyl hexosamine at up to seven sites of flagellin [71]. In the current dataset, this protein significantly accumulated in the membrane fraction after 90 min of nisin treatment (3-fold increase in abundance) and the expression of its coding gene has also been shown to be increased in a *sigH* mutant [44]. Interestingly, the expression of the sugar responsive regulator RpiR, which has been found in altered amounts (about a 6-fold increase in abundance) during adaptation to nisin in this study, is known to link the metabolic response with virulence in *S. aureus* [63]. Indeed, for most gastrointestinal pathogens, including *C. difficile* 630∆*erm*, flagella and flagellum mediated motility are recognized as essential virulence factors [72] and there is even strong evidence that the expression of flagellar genes in *C. difficile* is coupled to toxin gene regulation [73]. Moreover, the flagellar cap FliD and the flagellin structural component FliC have been characterized to be involved in adherence to the gastrointestinal epithelial and hence promote host colonization [74]. For *C. difficile* strain 630Δ*erm* it was found that mutants in fliC and fliD adhered more strongly to Caco-2 cells than the wild-type [75,76]. Concordantly, both mutant strains seem to be more virulent in hamsters [75]. In the case of nisin adaptation, this would mean that the enhanced expression of flagella proteins might not only contribute to nisin adaptation but could simultaneously also affect *C. difficile*’s virulence. Potentially, treatment with nisin would not only provide an antimicrobial effect but may also repress the virulence of the infecting strain. However, with the present state of knowledge, this remains speculative and awaits experimental validation.

### 4.3. Lipid II as Target for Antimicrobials

Lipid II, a central intermediate in peptidoglycan synthesis, is an ideal target for antimicrobial compounds such as nisin or vancomycin as its synthesis can be regarded a bottleneck in bacterial cell-wall synthesis. Indeed, lipid II significantly increases the affinity of nisin for the bacterial membrane [10,77], stabilizes the transmembrane orientation of nisin [78], and forms an integral part of the nisin pore [79]. The binding of the antibiotic vancomycin to lipid II obstructs the activity of penicillin-binding proteins (PBPs) to mature peptidoglycan and thus compromises the integrity of the cell envelope.

Whereas resistance to vancomycin involves the replacement of the lipid II pentapeptide with D-Ala-D-lac or D-Ala-D-Ser alternatives [80], this is not the case when cells are challenged with nisin. However, changes in the expression of proteins involved in peptidoglycan synthesis and membrane impairment have been observed after both vancomycin [81,82] and nisin treatment. Indeed, in *C. difficile* vancomycin resistance has been associated with mutations in MurG, which adds N-acetylglucosamine to lipid I to form lipid II [82]. Similarly, MurG accumulated up to 3.7-fold during adaptation of *C. difficile* to nisin, as observed in this study, supporting the assumption of a general role of MurG during adaptation and resistance development towards lipid II-targeting antimicrobials. On the other hand, it was found recently, that glucosamine-6-phosphate deaminase NagB (CDIF630erm_01147), glucose-6-phosphate isomerase Pgi (CDIF630erm_03585), both providing precursors for cell wall biosynthesis, and the peptidyl-prolyl cis-trans isomerase B PpiB (CDIF630erm_00459) involved in protein homeostasis of the cell envelope, exhibit a higher synthesis rate after vancomycin treatment in *C. difficile* [61]. In contrast, the analysis in this study revealed a lower amount of those proteins after nisin addition (up to 2.8-fold depletion), emphasizing unique features of each antimicrobial that are independent of the primary cellular target.

### 4.4. Bactericidal Activities of Various Membrane-Active Agents Against C. difficile

In bacteria, the function of the membrane is essential as it provides a selective barrier allowing active transport of nutrients and waste and maintenance of the cellular ion homeostasis [83].

The bactericidal effect of membrane-active antimicrobials was associated with two main mechanisms: (i) the dissipation of the transmembrane potential and/or the transmembrane pH leading to membrane depolarization [83,84,85] and (ii) the solubilization of parts of the bacterial membrane and membrane pore formation. The latter effect has also been summarized as a “detergent-like mechanism” as it enhances the membrane permeability to small molecules and was often correlated with the mode of action (MOA) of antimicrobial peptides such as nisin. However, the activity of detergents and antimicrobial peptides towards bacteria may differ depending on the composition of lipids on the cell surface and its net charge. Typically, antimicrobial peptides are more active and selective in their action than ordinary detergents, suggesting that there are principal differences as well [86].

For *C. difficile* it is tempting to compare the proteomic response to nisin to those towards bile acid, which also exhibits an amphiphilic structure and hence a saponaceous character. However, the correlation of the data presented in this study with those in the bile acid study published by Sievers et al. [53] was very low if it exists at all. Only 77 of all 792 proteins, which exhibited significant changes in protein abundance after nisin addition, showed comparable changes during bile acid stress. Although the time points analyzed and the level of growth inhibition was not completely comparable between this study and the study of Sievers et al. [53], it should still allow recognizing pattern of similar and specific regulation in response to treatment with the different agents. Indeed, the proteomic response to nisin seems to be specific to the antimicrobial action of this peptide and combats way more effects of nisin than just the detergent-like effect. However, in line with the saponaceous character of both, bile acids and nisin, the 77 proteins, which exhibits comparable changes in abundance in both datasets, are involved in the biosynthesis of the cell wall and cell surface components as well as in energy metabolisms, where especially the subunits of the ATP synthase were affected. Interestingly, there are also putative ABC-transporters and proteins with a predicted regulatory function, which show a comparable change in abundance after the addition nisin and bile acids, respectively. Unfortunately, only very little is known about the function of these specific proteins.

## Data Availability

All MS data were deposited to the ProteomeXchange Consortium via the PRIDE partner repository [48] with the dataset identifier PXD021684. All quantitative data on protein abundance can be found in Supplemental Tables S1 and S2.

## Supplementary Materials

The following are available online at https://www.mdpi.com/2073-4409/10/2/372/s1, Figure S1: Distribution of proteins to predicted subcellular localizations, Figure S2: Visualization of *C. difficile* 630∆*erm* flagella during adaptation to nisin, Table S1: Proteins quantified in the cytosolic fraction, Table S2: Proteins quantified in the membrane fraction.
