# Supplementary material for: Proteomic Adaptation of Clostridioides difficile to Treatment with the Antimicrobial Peptide Nisin
Source: Cells. 2021 Feb 11;10(2):372. doi: 10.3390/cells10020372 (PMC7918085; doi:10.3390/cells10020372)
Supplement: Supplementary file 1 [file cells-10-00372-s001.zip › Supplemental Figure S1.pdf]

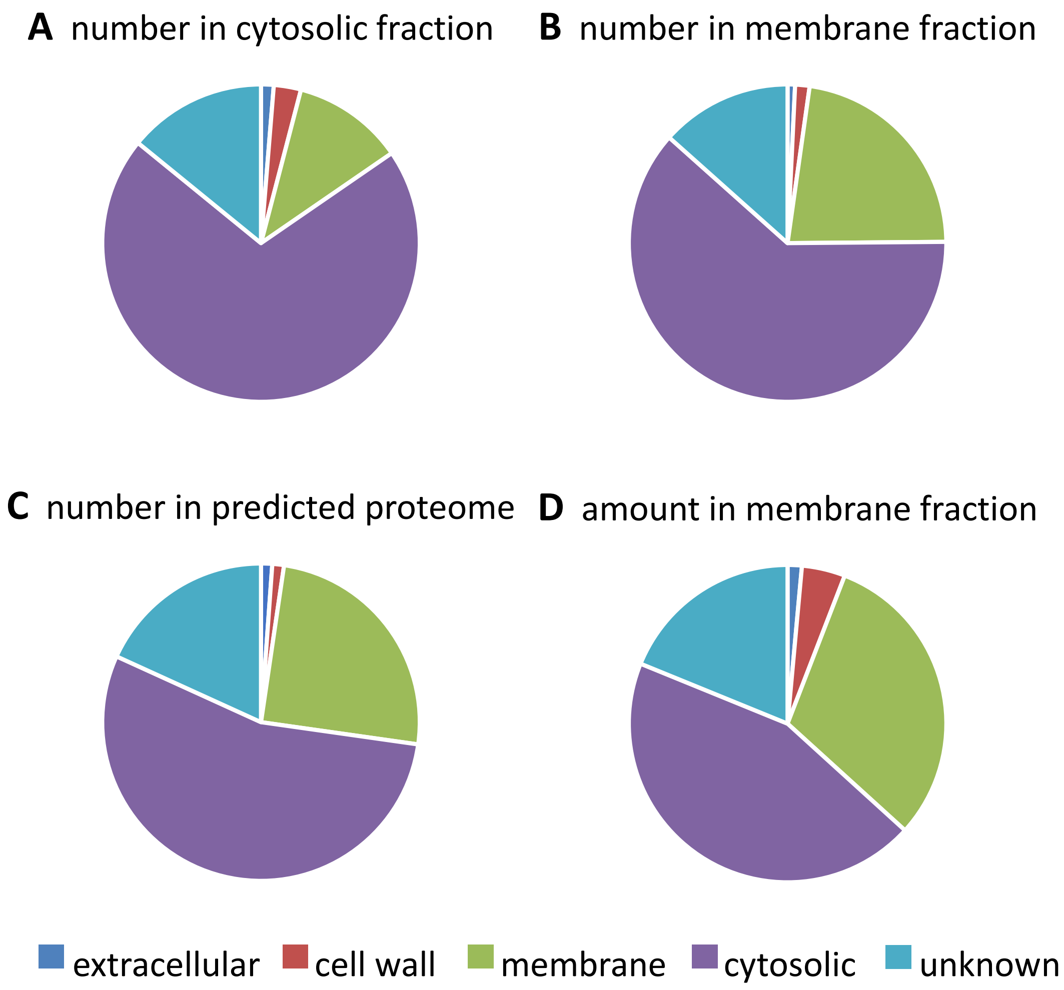

Figure S1: Distribution of proteins detected in this study to predicted subcellular localizations. Protein localization was predicted by psortB. The number of proteins assigned to the different subcellular compartments is shown for all proteins identified in either the cytosolic fraction (A) or the membrane enriched fraction (B) and compared to predicted localization of all proteins predicted in the proteome (C). In (D) the amount of proteins predicted for a specific localization was summed up to build the graph.
