# Supplementary material for: Proteomic Adaptation of Clostridioides difficile to Treatment with the Antimicrobial Peptide Nisin
Source: Cells. 2021 Feb 11;10(2):372. doi: 10.3390/cells10020372 (PMC7918085; doi:10.3390/cells10020372)
Supplement: Supplementary file 1 [file cells-10-00372-s001.zip › cells-1027148 Figure S2.pdf]

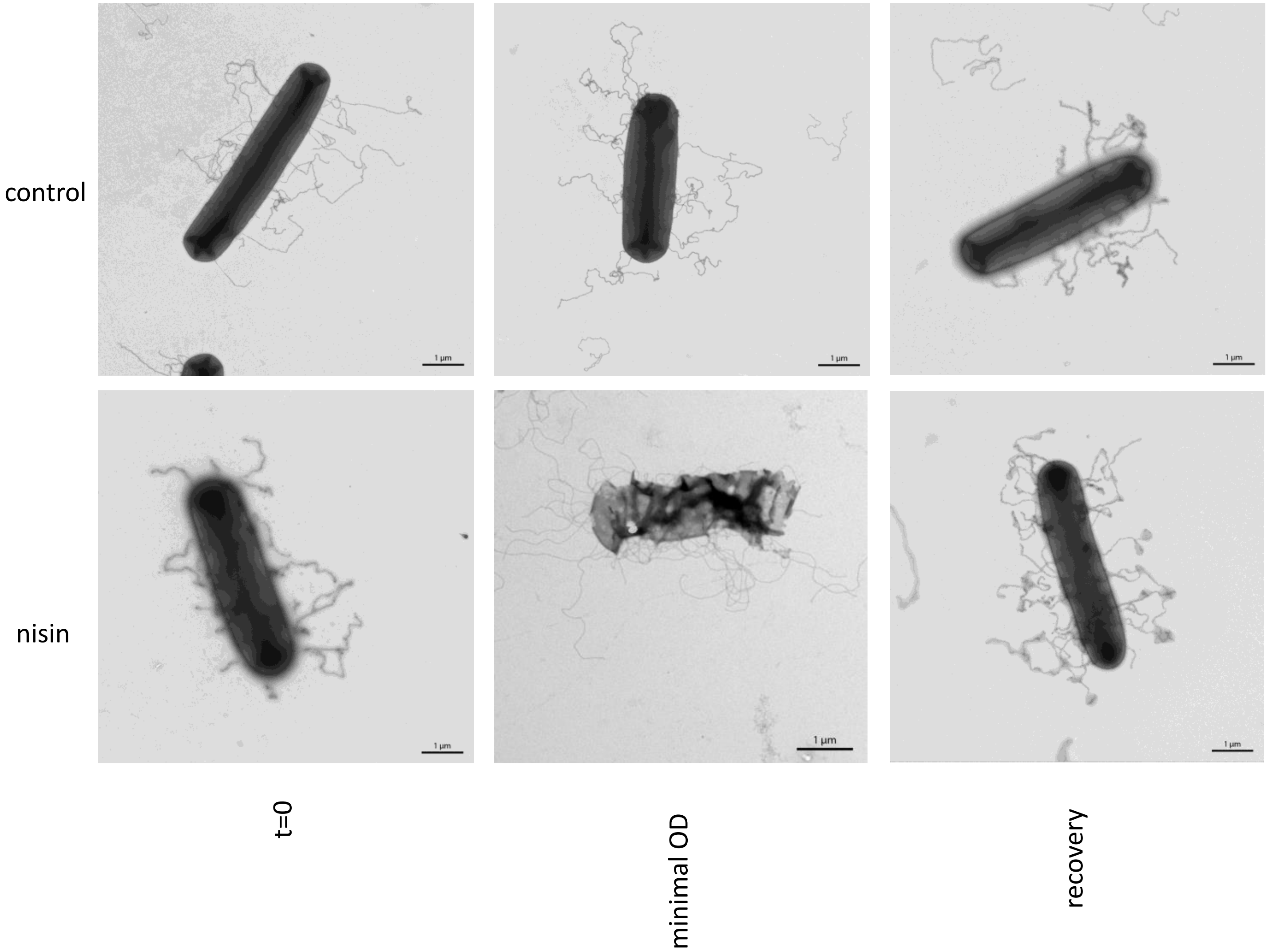

Supplemental Figure S2. Visualization of *C. difficile* 630Δ*erm* flagella during adaptation to nisin. Nisin was added to the cells displayed in line "nisin" whereas those displayed in line "control" were not treated. Samples were taken at the timepoint of nisin addition to the nisin culture (t=0), when the culture reached the lowest OD (minimal OD) and during the phase of growth recovery (recovery) (see Figure 1 for a representative growth curve). Representative images of negative stained cells are shown. Number of flagella per cell was determined for at least ten cells to obtain the data shown in Figure 8.
